# *N*-(3-Chloro­benzo­yl)-2-methyl­benzene­sulfonamide

**DOI:** 10.1107/S1600536811050574

**Published:** 2011-11-30

**Authors:** P. A. Suchetan, Sabine Foro, B. Thimme Gowda

**Affiliations:** a Department of Chemistry, Mangalore University, Mangalagangotri 574 199, Mangalore, India; b Institute of Materials Science, Darmstadt University of Technology, Petersenstrasse 23, D-64287, Darmstadt, Germany

## Abstract

In the title compound, C_14_H_12_ClNO_3_S, the N—H bond in the C—SO_2_—NH—C(O) segment is *anti* to the C=O bond. Further, the C=O bond and the *meta*-Cl atom in the benzoyl ring are also *anti* to each other. The dihedral angle between the sulfonyl and the benzoyl benzene rings is 72.4 (1)°. In the crystal, mol­ecules are linked by pairs of N—H⋯O hydrogen bonds, forming inversion dimers.

## Related literature

For our studies on the effects of substituents on the structures and other aspects of *N*-(ar­yl)-amides, see: Bowes *et al.* (2003 ▶); Gowda *et al.* (2004 ▶), on *N*-(ar­yl)-methane­sulfonamides, see: Jayalakshmi & Gowda (2004 ▶), on *N*-(ar­yl)-aryl­sulfonamides, see: Gowda *et al.* (2003 ▶), on *N*-(substitutedbenzo­yl)-aryl­sulfonamides, see: Suchetan *et al.* (2010 ▶) and on *N*-chloro­aryl­amides, see: Gowda *et al.* (1996 ▶).
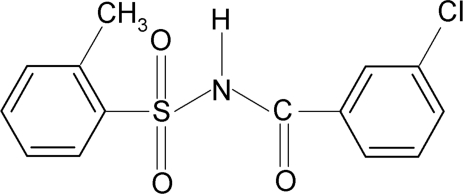


## Experimental

### 

#### Crystal data

C_14_H_12_ClNO_3_S
*M* *_r_* = 309.76
Monoclinic, 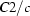

*a* = 18.043 (2) Å
*b* = 12.046 (1) Å
*c* = 15.596 (2) Å
β = 118.77 (2)°
*V* = 2971.3 (6) Å^3^
*Z* = 8
Mo *K*α radiation
μ = 0.40 mm^−1^
*T* = 293 K
0.40 × 0.36 × 0.32 mm

#### Data collection

Oxford Diffraction Xcalibur diffractometer with Sapphire CCD detector
Absorption correction: multi-scan (*CrysAlis RED*; Oxford Diffraction, 2009 ▶) *T*_min_ = 0.856, *T*_max_ = 0.882
5996 measured reflections
3024 independent reflections
2463 reflections with *I* > 2σ(*I*)
*R*_int_ = 0.012

#### Refinement

*R*[*F* ^2^ > 2σ(*F* ^2^)] = 0.042
*wR*(*F* ^2^) = 0.113
*S* = 1.03
3024 reflections
185 parameters
1 restraint
H atoms treated by a mixture of independent and constrained refinement
Δρ_max_ = 0.45 e Å^−3^
Δρ_min_ = −0.64 e Å^−3^

### 

Data collection: *CrysAlis CCD* (Oxford Diffraction, 2009 ▶); cell refinement: *CrysAlis CCD*); data reduction: *CrysAlis RED* (Oxford Diffraction, 2009 ▶; program(s) used to solve structure: *SHELXS97* (Sheldrick, 2008 ▶); program(s) used to refine structure: *SHELXL97* (Sheldrick, 2008 ▶); molecular graphics: *PLATON* (Spek, 2009 ▶); software used to prepare material for publication: *SHELXL97*.

## Supplementary Material

Crystal structure: contains datablock(s) I, global. DOI: 10.1107/S1600536811050574/bt5728sup1.cif

Structure factors: contains datablock(s) I. DOI: 10.1107/S1600536811050574/bt5728Isup2.hkl

Supplementary material file. DOI: 10.1107/S1600536811050574/bt5728Isup3.cml

Additional supplementary materials:  crystallographic information; 3D view; checkCIF report

## Figures and Tables

**Table 1:** Hydrogen-bond geometry (Å, °)

*D*—H⋯*A*	*D*—H	H⋯*A*	*D*⋯*A*	*D*—H⋯*A*
N1—H1*N*⋯O2^i^	0.82 (2)	2.06 (2)	2.876 (2)	174 (2)

Symmetry code: (i) 
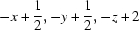
.

**Fig. 1.**
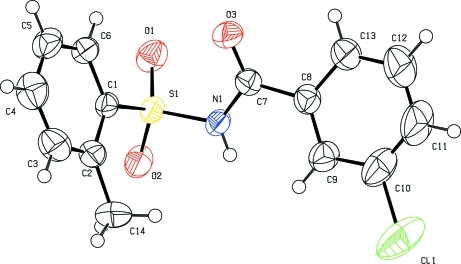
Molecular structure of the title compound, showing the atom-labelling scheme. Displacement ellipsoids are drawn at the 50% probability level.

**Fig. 2.**
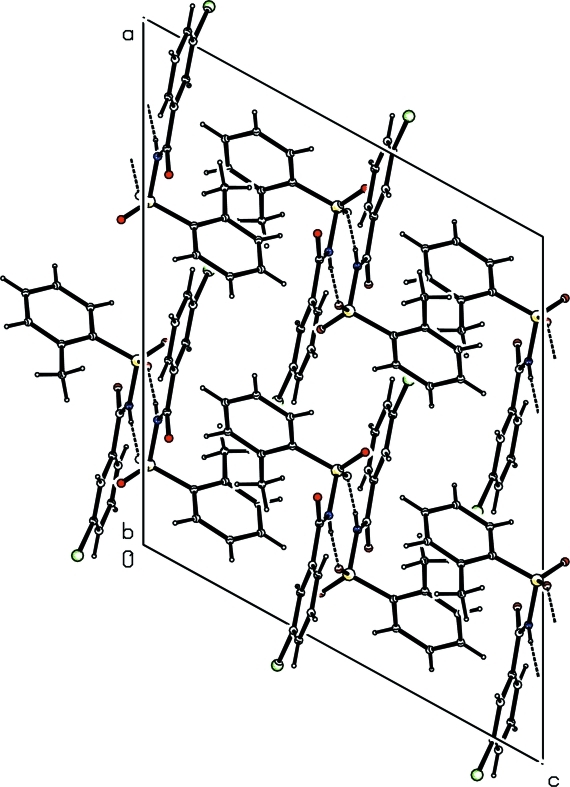
Molecular packing in the title compound. Hydrogen bonds are shown as dashed lines.

## supplementary crystallographic information

### Comment

Diaryl acylsulfonamides are known as potent antitumor agents against a broad spectrum of human tumor xenografts in nude mice. As part of our studies on the substituent effects on the structures and other aspects of *N*-(aryl)-amides (Bowes *et al.*, 2003; Gowda *et al.*, 2004), *N*-(aryl)-methanesulfonamides (Jayalakshmi & Gowda, 2004), *N*-(aryl)-arylsulfonamides (Gowda *et al.*, 2003); *N*-(substitutedbenzoyl)-arylsulfonamides (Suchetan *et al.*, 2010) and *N*-chloro-arylsulfonamides (Gowda *et al.*, 1996), in the present work, the crystal structure of *N*-(3-Chlorobenzoyl)- 2-methylbenzenesulfonamide (I) has been determined (Fig.1).

The conformation of the N—H bond in the C—SO_2_—NH—C(O) segment is *anti* to the C=O bond (Fig.1), similar to that observed in *N*-(benzoyl)-2-methylbenzenesulfonamide (II)(Suchetan *et al.*, 2010). Further, the conformation between the C=O bond and the *meta*-Cl in the benzoyl ring is also *anti* to each other.

The molecules are twisted at the *S* atom with the torsional angle of 66.9 (2)°, compared to the value of 68.8 (4)° in (II).

The dihedral angle between the sulfonyl benzene ring and the —SO_2_—NH—C—O segment is 82.4 (1)°, compared to the value of 84.8 (1)° in (II). Furthermore, the dihedral angle between the sulfonyl and the benzoyl benzene rings is 72.4 (1)°, compared to the value of 73.9 (1)° in (II).

The packing of molecules linked by of N—H···O(S) hydrogen bonds(Table 1) is shown in Fig. 2.

### Experimental

The title compound was prepared by refluxing a mixture of 3-chlorobenzoic acid (0.02 mole), 2-methylbenzenesulfonamide (0.02 mole) and excess phosphorous oxy chloride for 3 h on a water bath. The resultant mixture was cooled and poured into crushed ice. The solid, *N*-(3-chlorobenzoyl)2-methylbenzenesulfonamide, obtained was filtered, washed thoroughly with water and then dissolved in sodium bicarbonate solution. The compound was later reprecipitated by acidifying the filtered solution with dilute HCl. It was filtered, dried and recrystallized.

Prism like colourless single crystals of the title compound used in X-ray diffraction studies were obtained by slow evaporation of its toluene solution at room temperature.

### Refinement

The H atom of the NH group was located in a difference map and later restrained to N—H = 0.86 (2) %A. The other H atoms were positioned with idealized geometry using a riding model with C—H distances of 0.93Å (C-aromatic) and 0.96Å (C-methyl).

All H atoms were refined with isotropic displacement parameters were set at 1.2 *U*_eq_(C-aromatic, N) and 1.5 *U*_eq_(C-methyl).

### Crystal data

**Table:**

C_14_H_12_ClNO_3_S	*F*(000) = 1280
*M**_r_* = 309.76	*D*_x_ = 1.385 Mg m^−^^3^
Monoclinic, *C*2/*c*	Mo *K*α radiation, λ = 0.71073 Å
Hall symbol: -C 2yc	Cell parameters from 3176 reflections
*a* = 18.043 (2) Å	θ = 2.8–27.8°
*b* = 12.046 (1) Å	µ = 0.40 mm^−^^1^
*c* = 15.596 (2) Å	*T* = 293 K
β = 118.77 (2)°	Prism, colourless
*V* = 2971.3 (6) Å^3^	0.40 × 0.36 × 0.32 mm
*Z* = 8	

### Data collection

**Table:**

Oxford Diffraction Xcalibur diffractometer with Sapphire CCD detector	3024 independent reflections
Radiation source: fine-focus sealed tube	2463 reflections with *I* > 2σ(*I*)
graphite	*R*_int_ = 0.012
Rotation method data acquisition using ω and phi scans.	θ_max_ = 26.4°, θ_min_ = 2.9°
Absorption correction: multi-scan (*CrysAlis RED*; Oxford Diffraction, 2009)	*h* = −22→18
*T*_min_ = 0.856, *T*_max_ = 0.882	*k* = −15→11
5996 measured reflections	*l* = −18→19

### Refinement

**Table:**

Refinement on *F*^2^	Primary atom site location: structure-invariant direct methods
Least-squares matrix: full	Secondary atom site location: difference Fourier map
*R*[*F*^2^ > 2σ(*F*^2^)] = 0.042	Hydrogen site location: inferred from neighbouring sites
*wR*(*F*^2^) = 0.113	H atoms treated by a mixture of independent and constrained refinement
*S* = 1.03	*w* = 1/[σ^2^(*F*_o_^2^) + (0.0488*P*)^2^ + 4.0385*P*] where *P* = (*F*_o_^2^ + 2*F*_c_^2^)/3
3024 reflections	(Δ/σ)_max_ < 0.001
185 parameters	Δρ_max_ = 0.45 e Å^−^^3^
1 restraint	Δρ_min_ = −0.64 e Å^−^^3^

### Special details

**Table:**

Geometry. All e.s.d.'s (except the e.s.d. in the dihedral angle between two l.s. planes) are estimated using the full covariance matrix. The cell e.s.d.'s are taken into account individually in the estimation of e.s.d.'s in distances, angles and torsion angles; correlations between e.s.d.'s in cell parameters are only used when they are defined by crystal symmetry. An approximate (isotropic) treatment of cell e.s.d.'s is used for estimating e.s.d.'s involving l.s. planes.
Refinement. Refinement of *F*^2^ against ALL reflections. The weighted *R*-factor *wR* and goodness of fit *S* are based on *F*^2^, conventional *R*-factors *R* are based on *F*, with *F* set to zero for negative *F*^2^. The threshold expression of *F*^2^ > σ(*F*^2^) is used only for calculating *R*-factors(gt) *etc*. and is not relevant to the choice of reflections for refinement. *R*-factors based on *F*^2^ are statistically about twice as large as those based on *F*, and *R*- factors based on ALL data will be even larger.

### Fractional atomic coordinates and isotropic or equivalent isotropic displacement parameters (Å^2^)

**Table:**

	*x*	*y*	*z*	*U*_iso_*/*U*_eq_	
C1	0.34287 (11)	0.11403 (15)	0.87322 (14)	0.0349 (4)	
C2	0.29479 (13)	0.18523 (18)	0.79536 (16)	0.0449 (5)	
C3	0.30275 (17)	0.1707 (2)	0.71180 (18)	0.0604 (6)	
H3	0.2716	0.2161	0.6580	0.072*	
C4	0.35502 (18)	0.0918 (2)	0.70565 (19)	0.0627 (7)	
H4	0.3591	0.0854	0.6486	0.075*	
C5	0.40121 (16)	0.0224 (2)	0.78294 (19)	0.0546 (6)	
H5	0.4363	−0.0312	0.7785	0.065*	
C6	0.39508 (13)	0.03297 (18)	0.86754 (16)	0.0434 (5)	
H6	0.4257	−0.0139	0.9203	0.052*	
C7	0.22402 (13)	−0.03082 (17)	0.94079 (16)	0.0418 (5)	
C8	0.13770 (14)	−0.06030 (18)	0.92343 (16)	0.0451 (5)	
C9	0.07257 (14)	0.0159 (2)	0.89365 (18)	0.0535 (6)	
H9	0.0814	0.0905	0.8859	0.064*	
C10	−0.00629 (16)	−0.0220 (3)	0.8757 (2)	0.0736 (8)	
C11	−0.0197 (2)	−0.1309 (3)	0.8889 (3)	0.0970 (12)	
H11	−0.0729	−0.1545	0.8770	0.116*	
C12	0.0461 (2)	−0.2054 (3)	0.9201 (3)	0.0981 (12)	
H12	0.0372	−0.2793	0.9298	0.118*	
C13	0.12381 (17)	−0.1717 (2)	0.9368 (2)	0.0633 (7)	
H13	0.1678	−0.2227	0.9571	0.076*	
C14	0.23804 (18)	0.2758 (2)	0.7967 (2)	0.0657 (7)	
H14A	0.2719	0.3353	0.8380	0.079*	
H14B	0.2025	0.2469	0.8214	0.079*	
H14C	0.2036	0.3032	0.7314	0.079*	
N1	0.24872 (11)	0.07910 (15)	0.96409 (14)	0.0425 (4)	
H1N	0.2195 (14)	0.1279 (17)	0.9688 (18)	0.051*	
O1	0.40644 (10)	0.05491 (15)	1.05526 (11)	0.0546 (4)	
O2	0.34229 (10)	0.23938 (13)	1.01017 (13)	0.0547 (4)	
O3	0.27146 (10)	−0.09871 (13)	0.93618 (14)	0.0578 (4)	
Cl1	−0.08922 (5)	0.07097 (11)	0.83538 (9)	0.1187 (4)	
S1	0.34256 (3)	0.12455 (4)	0.98556 (4)	0.03864 (16)	

### Atomic displacement parameters (Å^2^)

**Table:**

	*U*^11^	*U*^22^	*U*^33^	*U*^12^	*U*^13^	*U*^23^
C1	0.0307 (9)	0.0335 (10)	0.0424 (10)	−0.0031 (8)	0.0192 (8)	−0.0026 (8)
C2	0.0401 (11)	0.0408 (11)	0.0523 (12)	0.0002 (9)	0.0210 (10)	0.0047 (10)
C3	0.0678 (16)	0.0615 (15)	0.0487 (13)	0.0021 (13)	0.0255 (12)	0.0117 (12)
C4	0.0773 (18)	0.0704 (17)	0.0510 (14)	−0.0068 (14)	0.0393 (13)	−0.0077 (13)
C5	0.0567 (14)	0.0533 (14)	0.0659 (15)	0.0006 (11)	0.0393 (12)	−0.0115 (12)
C6	0.0397 (11)	0.0412 (11)	0.0517 (12)	0.0025 (9)	0.0239 (9)	−0.0007 (9)
C7	0.0449 (11)	0.0368 (11)	0.0473 (11)	−0.0006 (9)	0.0251 (10)	−0.0003 (9)
C8	0.0448 (12)	0.0423 (12)	0.0512 (12)	−0.0074 (9)	0.0254 (10)	−0.0072 (10)
C9	0.0428 (12)	0.0514 (13)	0.0631 (14)	−0.0037 (10)	0.0229 (11)	−0.0074 (11)
C10	0.0432 (14)	0.092 (2)	0.0811 (19)	−0.0048 (14)	0.0263 (13)	−0.0196 (17)
C11	0.0587 (18)	0.090 (2)	0.149 (3)	−0.0355 (18)	0.055 (2)	−0.034 (2)
C12	0.093 (3)	0.0636 (19)	0.155 (4)	−0.0324 (19)	0.073 (3)	−0.015 (2)
C13	0.0608 (15)	0.0442 (13)	0.092 (2)	−0.0093 (12)	0.0422 (15)	−0.0075 (13)
C14	0.0622 (16)	0.0524 (14)	0.0783 (18)	0.0205 (13)	0.0304 (14)	0.0163 (13)
N1	0.0388 (9)	0.0374 (9)	0.0605 (11)	−0.0007 (7)	0.0312 (9)	−0.0054 (8)
O1	0.0428 (8)	0.0729 (11)	0.0445 (8)	0.0079 (8)	0.0182 (7)	0.0070 (8)
O2	0.0522 (9)	0.0466 (9)	0.0766 (11)	−0.0129 (7)	0.0401 (9)	−0.0226 (8)
O3	0.0557 (10)	0.0395 (8)	0.0884 (13)	0.0041 (7)	0.0428 (9)	−0.0047 (8)
Cl1	0.0500 (4)	0.1486 (10)	0.1396 (9)	0.0240 (5)	0.0313 (5)	−0.0175 (7)
S1	0.0337 (3)	0.0404 (3)	0.0455 (3)	−0.0024 (2)	0.0219 (2)	−0.0056 (2)

### Geometric parameters (Å, °)

**Table:**

C1—C6	1.389 (3)	C9—C10	1.388 (3)
C1—C2	1.396 (3)	C9—H9	0.9300
C1—S1	1.759 (2)	C10—C11	1.368 (5)
C2—C3	1.389 (3)	C10—Cl1	1.727 (3)
C2—C14	1.503 (3)	C11—C12	1.376 (5)
C3—C4	1.375 (4)	C11—H11	0.9300
C3—H3	0.9300	C12—C13	1.359 (4)
C4—C5	1.370 (4)	C12—H12	0.9300
C4—H4	0.9300	C13—H13	0.9300
C5—C6	1.382 (3)	C14—H14A	0.9600
C5—H5	0.9300	C14—H14B	0.9600
C6—H6	0.9300	C14—H14C	0.9600
C7—O3	1.211 (2)	N1—S1	1.6532 (17)
C7—N1	1.389 (3)	N1—H1N	0.816 (16)
C7—C8	1.490 (3)	O1—S1	1.4170 (17)
C8—C9	1.384 (3)	O2—S1	1.4361 (16)
C8—C13	1.399 (3)		
			
C6—C1—C2	122.21 (19)	C11—C10—C9	121.5 (3)
C6—C1—S1	116.20 (16)	C11—C10—Cl1	119.5 (2)
C2—C1—S1	121.57 (15)	C9—C10—Cl1	119.0 (3)
C3—C2—C1	115.9 (2)	C10—C11—C12	119.6 (3)
C3—C2—C14	118.8 (2)	C10—C11—H11	120.2
C1—C2—C14	125.2 (2)	C12—C11—H11	120.2
C4—C3—C2	122.4 (2)	C13—C12—C11	120.5 (3)
C4—C3—H3	118.8	C13—C12—H12	119.7
C2—C3—H3	118.8	C11—C12—H12	119.7
C5—C4—C3	120.5 (2)	C12—C13—C8	120.0 (3)
C5—C4—H4	119.8	C12—C13—H13	120.0
C3—C4—H4	119.8	C8—C13—H13	120.0
C4—C5—C6	119.3 (2)	C2—C14—H14A	109.5
C4—C5—H5	120.3	C2—C14—H14B	109.5
C6—C5—H5	120.3	H14A—C14—H14B	109.5
C5—C6—C1	119.6 (2)	C2—C14—H14C	109.5
C5—C6—H6	120.2	H14A—C14—H14C	109.5
C1—C6—H6	120.2	H14B—C14—H14C	109.5
O3—C7—N1	120.84 (19)	C7—N1—S1	122.50 (14)
O3—C7—C8	122.37 (19)	C7—N1—H1N	124.8 (18)
N1—C7—C8	116.78 (18)	S1—N1—H1N	112.7 (18)
C9—C8—C13	120.2 (2)	O1—S1—O2	118.13 (11)
C9—C8—C7	123.2 (2)	O1—S1—N1	109.57 (10)
C13—C8—C7	116.6 (2)	O2—S1—N1	103.78 (9)
C8—C9—C10	118.1 (2)	O1—S1—C1	109.27 (9)
C8—C9—H9	120.9	O2—S1—C1	109.73 (10)
C10—C9—H9	120.9	N1—S1—C1	105.56 (9)
			
C6—C1—C2—C3	−0.5 (3)	C8—C9—C10—Cl1	−178.3 (2)
S1—C1—C2—C3	177.92 (17)	C9—C10—C11—C12	−0.6 (6)
C6—C1—C2—C14	−179.0 (2)	Cl1—C10—C11—C12	179.4 (3)
S1—C1—C2—C14	−0.6 (3)	C10—C11—C12—C13	−0.6 (6)
C1—C2—C3—C4	−0.4 (4)	C11—C12—C13—C8	0.7 (6)
C14—C2—C3—C4	178.2 (3)	C9—C8—C13—C12	0.3 (4)
C2—C3—C4—C5	0.8 (4)	C7—C8—C13—C12	−178.5 (3)
C3—C4—C5—C6	−0.3 (4)	O3—C7—N1—S1	0.7 (3)
C4—C5—C6—C1	−0.5 (3)	C8—C7—N1—S1	179.90 (15)
C2—C1—C6—C5	0.9 (3)	C7—N1—S1—O1	−50.7 (2)
S1—C1—C6—C5	−177.54 (17)	C7—N1—S1—O2	−177.71 (18)
O3—C7—C8—C9	−156.0 (2)	C7—N1—S1—C1	66.88 (19)
N1—C7—C8—C9	24.8 (3)	C6—C1—S1—O1	7.75 (19)
O3—C7—C8—C13	22.7 (3)	C2—C1—S1—O1	−170.73 (16)
N1—C7—C8—C13	−156.5 (2)	C6—C1—S1—O2	138.74 (16)
C13—C8—C9—C10	−1.4 (4)	C2—C1—S1—O2	−39.75 (19)
C7—C8—C9—C10	177.3 (2)	C6—C1—S1—N1	−110.00 (16)
C8—C9—C10—C11	1.6 (4)	C2—C1—S1—N1	71.51 (18)

### Hydrogen-bond geometry (Å, °)

**Table:**

*D*—H···*A*	*D*—H	H···*A*	*D*···*A*	*D*—H···*A*
N1—H1N···O2^i^	0.82 (2)	2.06 (2)	2.876 (2)	174 (2)

Symmetry codes: (i) −*x*+1/2, −*y*+1/2, −*z*+2.
